# Methotrexate Shortages and Their Impact on Chronic Inflammatory Rheumatic Disease Management: Results From a National Survey of Moroccan Rheumatologists

**DOI:** 10.7759/cureus.82640

**Published:** 2025-04-20

**Authors:** Hind L'Heri, Hanan Rkain, Imane Bensaghir, Latifa Tahiri, Jihane Belayachi, Redouane Abouqal, Hajjaj-Hassouni Najia, Fadoua Allali

**Affiliations:** 1 Department of Rheumatology B, Ayachi Hospital, Ibn Sina Hospital Center, Faculty of Medicine and Pharmacy, Mohammed V University, Rabat, MAR; 2 Exercise Physiology and Autonomous Nervous System Team, Physiology Laboratory, Faculty of Medicine and Pharmacy, Mohammed V University, Rabat, MAR; 3 Acute Medical Unit, Ibn Sina University Hospital, Rabat, MAR; 4 Laboratory of Biostatistics, Clinical and Epidemiological Research, Faculty of Medicine and Pharmacy, Mohammed V University, Rabat, MAR; 5 International University of Rabat (UIR), College of Health Sciences, Research Center of Health Sciences (CreSS), Rabat, MAR

**Keywords:** chronic inflammatory rheumatic disease, impact, management, methotrexate shortages, rheumatologists

## Abstract

Objective: To assess the impact of methotrexate (MTX) shortages on the management of chronic inflammatory rheumatic diseases (CIRD) in Morocco and their consequences on patient care.

Material and methods: We conducted a cross-sectional online survey among Moroccan rheumatologists. A structured Google Forms (Google Inc., Mountain View, CA, USA) questionnaire was emailed to rheumatologists, divided into five sections: sociodemographic data, MTX indications and prescription methods, patient-reported reasons for stopping MTX, consequences of stopping MTX, and possible solutions.

Results: The survey was completed by 153 rheumatologists (mean age 42.9 ± 12.7 years, 84.8% female, mean length of practice 13.8±11 years). Overall, 98.7% considered MTX as the conventional disease-modifying antirheumatic drug (cDMARD) of choice for treating CIRD, with 84.3% prescribing it primarily for rheumatoid arthritis. The main reason for MTX discontinuation was unavailability of treatment (96.1%), and 97.4% of rheumatologists reported difficulties in prescribing due to recurrent stock shortages. Consequences included increased use of higher daily doses of oral corticosteroids (94%), higher rates of hospitalization for corticosteroid bolus infusions (79%), therapeutic escalation to biologics in biologic-naïve patients (67.3%), and reduced effectiveness of current biologic therapy (44.6%).

Conclusion: This survey highlights the significant impact of MTX shortages on CIRD management, driving reliance on expensive biologics and deteriorating patients’ and families’ quality of life. Urgent action is needed to ensure a continuous MTX supply.

## Introduction

Methotrexate (MTX) is widely recommended as the first-line treatment for chronic inflammatory rheumatic diseases, including rheumatoid arthritis (RA) [1], psoriatic arthritis (PsA) [2] and juvenile idiopathic arthritis (JIA) [3]. Its proven efficacy and well-established safety profile make it a cornerstone in managing these diseases. MTX exerts its anti-inflammatory effects by modulating several immune pathways. It promotes the accumulation of adenosine, inhibits the activation of the NF-κB factor, sensitises T lymphocytes to apoptosis and regulates the expression of the non-coding RNA lincRNA-p21; these actions contribute to the reduction of inflammation and the prevention of joint damage [4-6].

MTX can be administered orally, subcutaneously, or, less commonly, intramuscularly [7]. The parenteral route, especially at high doses, improves bioavailability [8,9] and clinical efficacy compared with oral administration [7,9,10]. The subcutaneous route is often preferred due to better gastrointestinal tolerability, compliance and adherence [11].

The El Ayachi cohort followed patients with new-onset RA (<12 months) for two years with standard clinical, biological, functional and structural assessments; one-third of MTX monotherapy patients achieved remission [12].

Despite the advent of biologic therapies, MTX remains essential and is often combined with biologics to improve efficacy and avoid immunogenicity [1,13].

The efficacy of conventional disease-modifying antirheumatic drugs (cDMARDs) such as MTX depends on patient adherence [14,15]. Non-adherence, manifested as irregular or incorrect use of treatment, can lead to a reduction in the efficacy of MTX, worsening of symptoms and an increase in long-term complications. Factors contributing to non-adherence include not only the unavailability of MTX, but also the perception of side effects, the complexity of the drug regimen, economic constraints and various psychosocial aspects.

Non-adherence is associated with a significant medico-economic burden. It increases the number of consultations, hospitalisations and additional treatments, leading to higher healthcare costs, and adversely affects patients' quality of life by worsening their condition and limiting their daily activities.

Despite its well-established therapeutic benefits, recurrent MTX shortages are a real problem affecting compliance in chronic inflammatory rheumatic disease (CIRD) patients. In recent years, frequent shortages of this essential drug have increased internationally [16], particularly in Morocco, leading to poor compliance and compromising the optimal management of CIRDs.

The aim of this study is, firstly, to analyze the impact of the shortage of methotrexate on the therapeutic management of CIRDs in Morocco and, secondly, to gather the perceptions of Moroccan rheumatologists regarding its clinical consequences.

## Materials and methods

Study design and population

We conducted a national cross-sectional study, self-administered online, among rheumatologists practicing in Morocco, between January 10 and February 28, 2025.

Study eligibility criteria

All rheumatologists practising in Morocco were included, regardless of their sector of activity (public, private or university). No exclusion criteria were applied, in order to ensure maximum representation of the target population.

Sampling strategy

This was an exhaustive exploratory study, targeting all Moroccan rheumatologists. The questionnaire was distributed to all practitioners registered with the Moroccan Society of Rheumatology (SMR), which has a database containing the professional email addresses of 440 rheumatologists.

Data collection

The data was collected using the online survey tool Google Forms (Google Inc., Mountain View, CA, USA), ensuring easy access and efficient data collection. A list of 440 rheumatologists practicing in Morocco, provided by the Moroccan SMR, was used to distribute the questionnaire. An initial e-mail explaining the objective and context of the study, as well as a link to the survey, was sent on January 10, 2025. A reminder e-mail was sent on February 10, 2025 to increase participation. Only responses collected between January 10 and February 28, 2025 were included in the analysis. To guarantee the exhaustivity of the data, all answers were mandatory. The questionnaire was elaborated to explore prescription practices, obstacles encountered and consequences related to iterative breaks of MTX in patients with CRIDs in Morocco.

Questionnaire

The questionnaire attached in the appendix was drawn up by a group of five rheumatology experts working in university hospitals (doctorate and professor level), based on an exhaustive review of the literature and a reflection on the national issues related to access to MTX. Two sets of revisions were made to ensure the pertinence, clarity and adequacy of the questions with the objectives of the study.

A pilot phase was conducted with 10 rheumatologists to assess the clarity and pertinence of the questionnaire. This pre-test enabled the wording to be refined and clarity to be improved. The average time taken to complete the questionnaire was seven minutes.

The final version of the questionnaire was structured in five main sections as detailed below.

The first section collected demographic characteristics of the rheumatologists (age, gender, sector of practice and years of experience).

The second section evaluated MTX prescription practices, specifying therapeutic indications and routes of administration used.

The third section explored rheumatologists' perceptions of common reasons for MTX discontinuation in their patients.

The fourth section examined the consequences observed after MTX discontinuation, with six dichotomous (yes/no). questions.

The fifth section identified the main difficulties encountered by rheumatologists in prescribing MTX, as well as the measures deemed necessary to remedy these difficulties and improve access to treatment.

Statistical analysis

Data were analyzed using Jamovi 2.3.19 software. Qualitative variables were reported as numbers and percentages, quantitative variables as means ± standard deviations or medians [interquartile ranges], based on distribution. 

Ethical considerations

The study protocol was approved by the Biomedical Research Ethics Committee, Faculty of Medicine and Pharmacy, Mohammed V University, Rabat (approval number: 37/25), adhering to the 1964 Helsinki Declaration and its amendments. Participants were informed of the study’s purpose, procedures, and voluntary nature, providing informed consent. Anonymity was maintained.

## Results

Among the 440 questionnaires distributed, 153 rheumatologists responded (response rate: 34.7%). The mean age of the participants was 42.9 ± 12.7 years. Participants had a mean age of 42.9 ± 12.7 years, were predominantly female (84.8%), and had a median practice experience of 13.8 years [IQR: 6-22]. Practice sectors included private (39.2%), public (28.1%), residents (22.9%), and university hospitals (9.8%).

MTX was considered the cDMARD of choice for CIRDs by 98.7% of respondents, with 84.3% prescribing it always for rheumatoid arthritis. The subcutaneous route dominated administration (86.9%). See Table 1 for prescribing practices.

**Table 1:** Prescribing Practices for Methotrexate Among Moroccan Rheumatologists. cDMARD: conventional Disease-Modifying Anti-Rheumatic Drug

Practices	Effective (n)	Percentage (%)
I consider methotrexate the cDMARD of choice for the treatment of chronic inflammatory rheumatic diseases.	151	98.7
I always prescribe methotrexate for the following conditions:		
- Rheumatoid arthritis	129	84.3
- Psoriatic arthritis	97	63.3
- Peripheral spondyloarthritis	51	33.3
- Juvenile idiopathic arthritis	46	30.0
I always prescribe methotrexate in the following presentation forms:		
- Subcutaneous injection	133	86.9
- Intramuscular injection	52	33,9
- Oral administration	6	3.9

Rheumatologists identified unavailability (96.1%), gastrointestinal side effects (95.4%), high cost (81.7%), and lack of therapeutic education (64.2%) as reasons for patient discontinuation of MTX (Table 2). 

**Table 2:** Rheumatologists’ Perception Regarding Reasons of Methotrexate Discontinuation.

Reason	Effective (n)	Percentage (%)
Unavailability of treatment	147	96.1
Gastrointestinal side effects	145	95.4
High cost of methotrexate	125	81.7
Lack of therapeutic education	98	64.2
Fear of injection	53	35.1
Treatment ineffectiveness	46	30.1
Dermatological side effects	36	23.8

**Figure 1:**
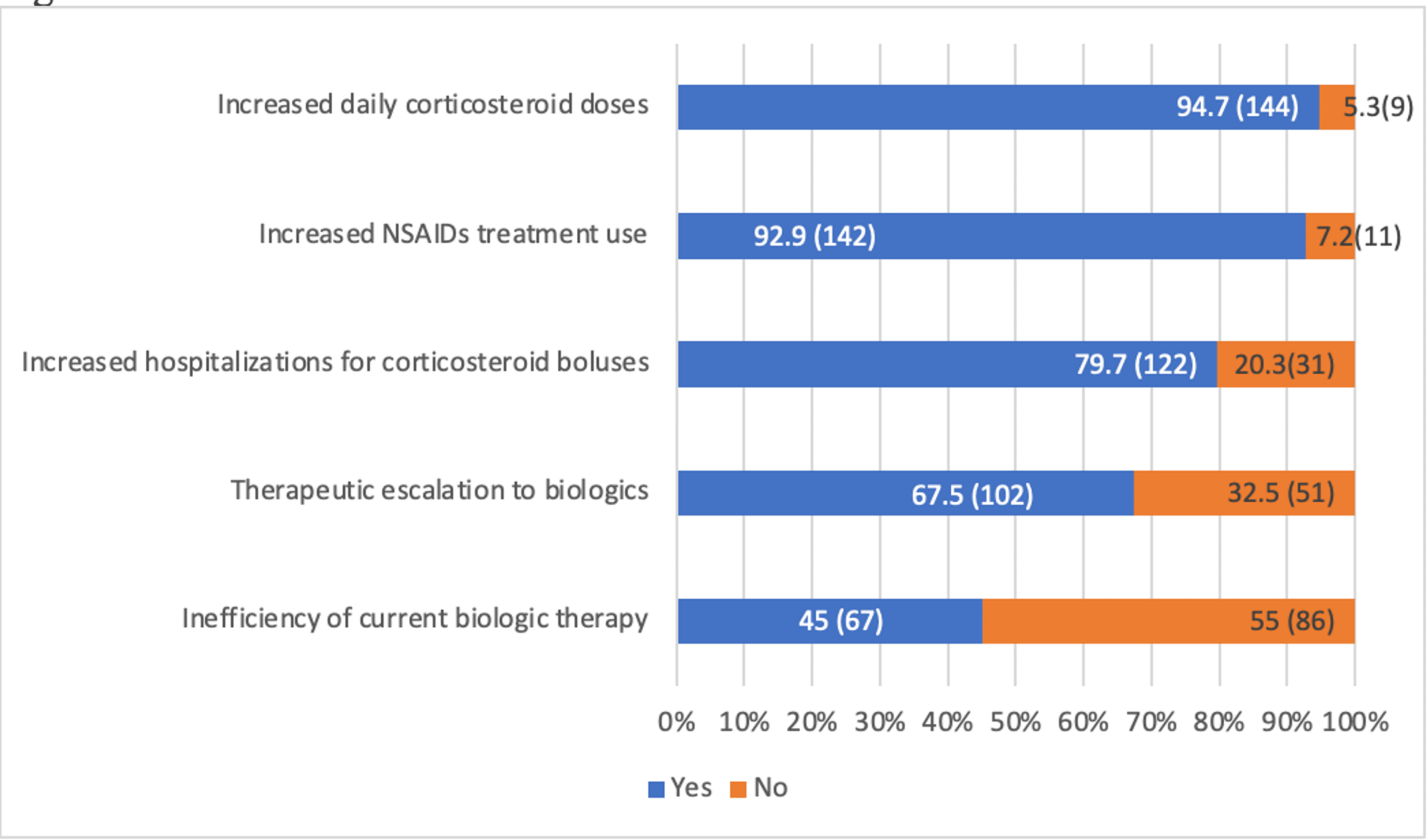
Consequences of Methotrexate Discontinuation on Chronic Inflammatory Rheumatic Disease (CIRD) Management NSAIDs: Non-Steroidal Anti-Inflammatory Drugs % (frequency=n): Values are expressed as percentages (%), with absolute frequencies (n) indicated in parentheses.

Consequences of MTX discontinuation included increased daily corticosteroid doses (94.7%), symptomatic treatment use (92.2%), hospitalizations for corticosteroid boluses (79.6%), escalation to biologics (67.3%), and reduced biologic efficacy (44.6%) (Figure 1).

All respondents (100%) agreed on the need to supply the oral form of methotrexate in Morocco, to lower its price, to reinforce therapeutic education for patients and to ensure that it is covered by the third-party payment system. They also insisted on the need to ensure the availability of this treatment in health centers (94.1%).

## Discussion

Our research highlights key challenges to the use of MTX in Morocco: non-adherence, prescribing barriers and the detrimental impact of supply shortages and cost constraints on the management of CIRDs.

Since its introduction in the 1980s, MTX has been a cornerstone of rheumatic disease management worldwide due to its proven efficacy and favourable safety profile. As such, it is recommended as first-line therapy by both the European League Against Rheumatism (EULAR) and the American College of Rheumatology (ACR) [17,18]. While MTX is generally well tolerated, it can cause side effects, including gastrointestinal, hepatic, hematologic, dermatologic, infectious, and renal disorders [19]. Although folic acid supplementation can reduce these adverse effects [20,21], they remain a significant cause of discontinuation.

In Morocco, MTX is only available in injectable form, which creates accessibility challenges and contributes to frequent shortages. Internationally, the oral formulation offers a more flexible treatment option. While a clinical trial by Braun et al. [22] showed similar tolerability between oral and parenteral MTX, the injectable route demonstrated slightly superior efficacy.

MTX is the cDMARD of choice for RA [1,4] and PsA [2], a view shared by 98.7% of Moroccan rheumatologists. However, the main reasons for discontinuation, as reported by respondents, are unavailability (96.1%), gastrointestinal side effects (95.4%) and high cost (81.7%).

Our rheumatologists propose the promotion of oral MTX in Morocco, citing improved patient compliance and cost reduction as demonstrated by international economic studies [23,24].

While the Moroccan Society of Rheumatology recommends MTX as a first-line treatment, reserving biologics for failure or intolerance [25], our study shows that the unavailability of MTX leads to the overuse of biologic therapy, resulting in a significant financial burden. This is highlighted by Fellous et al.'s study of the Moroccan rheumatoid arthritis registry, which reported annual biologic costs ranging from €1,472 to €9,879, resulting in a significant burden on the healthcare system [26].

Rheumatologists report that the unavailability of MTX has drastically reduced the efficacy of biologic treatments in almost 50% of patients. Furthermore, the resulting increase in hospitalisations for CIRD flares has necessitated the use of high-dose corticosteroids, which pose significant short- and long-term health risks. This critical situation has also significantly reduced the quality of life of patients and their families.

All rheumatologists urge rapid commercialisation of oral MTX, involvement of third-party payers and continued availability of primary care to stabilise the management of CIRDs. Improved patient education could further reduce discontinuation, reduce reliance on costly biologics and hospitalisations, and thus optimise healthcare resources.

This analysis highlights the significant economic and medical challenges faced by patients with CIRDs due to limited access to MTX. Improved adherence to treatment with MTX reduces the need for costly biotherapies and hospitalisations associated with disease flares.

Consequently, implementation of these interventions would improve patient quality of life and optimise healthcare resource use by reducing the direct and indirect costs of CIRD management. These recommendations, based on evidence-based principles, aim to ensure sustainable, accessible and economically viable care for this patient population.

Study limitations

Although this study offers valuable insights into the challenges of MTX in Morocco, it has limitations. First, the response rate may introduce non-response bias, which limits the generalisability of the results. In addition, we were unable to accurately quantify the additional costs associated with MTX shortages, particularly those related to biotherapy switching, increased corticosteroid use and hospitalisations. Further research is needed to fully assess the overall economic impact.

## Conclusions

The detrimental impact of methotrexate shortages on the management of CIRDs is clearly demonstrated by this study. Treatment interruptions lead to major clinical complications, drive the use of less cost-effective biotherapies and severely impact patients' quality of life. To mitigate these effects, ensuring a continuous supply of MTX, improving affordability and implementing therapeutic optimisation strategies are critical to both reducing healthcare costs and improving the care of patients with chronic inflammatory rheumatic diseases.

## Appendices

Socio-demographic data 

 Age:  ………… Years

 Gender:

 Women

 Man

 Length of service (in years): ............

 Practice status:

·       You are:

o    Rheumatology Resident

o    Rheumatologist in the public sector

o    Rheumatologist in the private sector

o    Rheumatologist in the CHU sector

Methotrexate prescribing practices 

In your practice, do you consider MTX to be the csDMARD of choice in RICs?

Yes

No

In your practice, you prescribe methotrexate in the following conditions:

Rheumatoid arthritis

Always

Often

Sometimes

Rarely 

Never

Peripheral form of spondyloarthritis

Always

Often

Sometimes

Rarely 

Never

Psoriatic arthritis

Always

Often

Sometimes

Rarely 

Never

Juvenile idiopathic arthritis

Always

Often

Sometimes

Rarely 

Never

In your practice, do you prescribe methotrexate in the form of:

Subcutaneous injectable 

Always

Often

Sometimes

Rarely 

Never

Intramuscular injectable form

Always

Often

Sometimes

Rarely 

Never

Oral form 

Always

Often

Sometimes

Rarely 

Never

 *Reasons for discontinuation of methotrexate (MTX)*

In your practice, what are the main reasons that push the RIC patient to stop MTX?

Yes

No

Ineffectiveness of treatment

Yes

No

Occurrence of digestive side effects

Yes

No

Appearance of side effects of the skin, hair...

Yes

No

Lack of therapeutic education

Yes

No

Fear of injection

Yes

No

Desire for pregnancy

Yes

No

High price of the drug

Yes

No

Unavailability of the drug

Yes

No

Consequences of MTX discontinuation

What were the consequences of the discontinuation of MTX, linked to the problems of shortages and/or price increases, on the care of your patients?

Increase in daily doses of corticosteroid therapy

Yes

No

Increase in the use of NSAIDs

Yes

No

Hospitalisation for Cortisonic Bolus perfusion

Yes

No

Switch to another csDMARD (leflunomide, salazopyrine, combination of csDMARDs)

Yes

No

Therapeutic escalation to biotherapy (for biotherapy-naïve patients)

Yes

No

Ineffectiveness of ongoing biotherapy (patients already on biotherapy)

Yes

No

Solutions to overcome MTX shortage challenges

Marketing of the oral form of MTX in Morocco

 Yes

No

Include MTX in the third-party payment system (as part of the ALD condition)

Yes

No

Ensuring the availability of MTX in health centers and diagnostic centers

 Yes

 No

Lower MTX price

 Yes

 No

Strengthening the therapeutic education of CIRDs patients on MTX

 Yes

No
